# Primary thoracic synovial sarcomas: clinical profile and treatment outcomes of a rare entity managed at a tertiary care centre

**DOI:** 10.3332/ecancer.2024.1757

**Published:** 2024-09-06

**Authors:** Ghazal Tansir, Sameer Rastogi, Ekta Dhamija, Shamim Ahmed Shamim, Deepali Jain, Adarsh Barwad, Sunil Kumar, Rambha Pandey

**Affiliations:** 1 Department of Medical Oncology, All India Institute of Medical Sciences, New Delhi 110029, India; 2 Department of Radiodiagnosis, All India Institute of Medical Sciences, New Delhi 110029, India; 3 Department of Nuclear Medicine, All India Institute of Medical Sciences, New Delhi 110029, India; 4 Department of Pathology, All India Institute of Medical Sciences, New Delhi 110029, India; 5 Department of Surgical Oncology, All India Institute of Medical Sciences, New Delhi 110029, India; 6 Department of Radiation Oncology, All India Institute of Medical Sciences, New Delhi 110029, India

**Keywords:** synovial sarcoma, multidisciplinary treatment, primary thoracic sarcoma, thoracic oncology

## Abstract

**Introduction:**

Primary thoracic synovial sarcoma (PTSS) is a rare malignancy presenting with varying clinical manifestations. There is a paucity of data with few studies dedicated to this unique subset of neoplasms. We present our findings from one of the largest real-world studies among patients with PTSS.

**Methods:**

This is a single-centre, real-world study in patients with PTSS included between 2017 and 2023. Survival estimates were obtained by the Kaplan-Meier method and Cox regression analysis.

**Results:**

24 patients with a median age of 34.5 years (range 16–54) presented with chest pain (*n* = 11, 45.8%) and dyspnea (*n* = 10, 41.6%). Predominant primary sites of disease were the lung (*n* = 12, 50%) and mediastinum (*n* = 6, 25%). The stage at presentation was unresectable locally advanced (*n* = 10, 41.6%), localised (*n* = 8, 33.3%) and metastatic (*n* = 6, 25%) with pulmonary metastases (*n* = 10, 62.5%) and pleural effusion (*n* = 4, 25%). 16 (66.6%) patients underwent surgical resection including 7 (43.8%) who received neoadjuvant chemotherapy (NACT). NACT was given in ten patients producing stable disease in 5 (50%) and partial response in 3 (30%) patients, respectively, with surgery performed in 7 (70%). 11 (62.5%) operated patients had a microscopically complete resection and 10 (41.6%) received postoperative radiotherapy. Anthracyclines were given in 23 (95.8%) patients in the first line, while pazopanib was the most common therapy in the second and third lines, respectively. At a median follow-up of 32 months (range 16.7–47.2), the median overall survival (OS) was 41 months (95% CI: 23.7–58.2) and 8 months (95% CI: 1–25.6) overall and in metastatic disease, respectively. Presentation with metastases (*p* = 0.01) and treatment with surgical resection (*p* = 0.005) were significantly associated with OS on univariate analysis.

**Interpretation:**

The locally advanced nature of the disease at presentation signifies the need for early diagnosis and technically superior definitive therapies. The survival outcomes for metastatic disease remain poor and the need for novel therapies for advanced disease remains unmet so far.

**Clinical trial registration:**

Not applicable

## Background

Primary thoracic sarcomas constitute a rare subset of thoracic neoplasms. Pulmonary and chest wall sarcomas constitute 0.5% and 5% of all thoracic malignancies, respectively [1, 2]. While synovial sarcoma (SS) typically occurs at the extremities and trunk, primary thoracic synovial sarcoma (PTSS) has been described as less than 2% of all SS [3]. The primary subsites included among PTSS are the chest wall, mediastinum, lung and heart. Due to the involvement of vital mediastinal structures, clinical presentations may include respiratory and cardiac symptoms. Multidisciplinary treatment (MDT) includes surgery for resectable disease, with or without radiotherapy (RT) and adjuvant chemotherapy. Resection margin status and tumour size have been found to be important prognostic factors for localised disease [4]. Expertise in surgical resection and RT planning is paramount in cases of PTSS. Locally advanced or metastatic disease is managed with doxorubicin-based chemotherapeutic regimens, while subsequent therapies for advanced and metastatic disease are less well-defined. We conducted this retrospective study among patients with PTSS to examine the clinical characteristics, treatment patterns and outcomes of this rare disease.

## Methods

This is a single-centre retrospective analysis of a prospectively maintained database evaluating patients with PTSS. The patients included in the study were registered in the sarcoma medical oncology clinic of All India Institute of Medical Sciences between May 2017 and January 2023. After clearance from the Institute Review Board, patient data were evaluated through hospital records including epidemiologic characteristics, stage of the disease, primary and metastatic sites, therapy administered, response rates and outcomes.

Chest wall, mediastinal, pulmonary and cardiac sites were included in the study and staged as localised, locally advanced unresectable and metastatic disease. Staging as locally advanced unresectable disease was deemed as per the expert thoracic surgeons in the multidisciplinary tumour board meetings. The histopathological diagnosis of SS had been reviewed by the expert sarcoma pathologists (AB and AM) at our institution. Statistical analysis was done through SPSS 26 (SPSS, Chicago, IL). Nominal data were entered as numbers (%) and continuous data as median and mean values as applicable. Progression-free survival (PFS) was calculated from the date of initiation of treatment to the first date of documented progressive disease or death from any cause. Overall survival (OS) was calculated by the Kaplan-Meier method from the date of diagnosis to death from any cause; patients alive or lost to follow up were censored. Prognostic variables were analysed for association with OS and PFS by the chi square test and multivariate analysis was carried out by Cox regression analysis.

## Results

24 patients with PTSS were included with a median age of 34.5 years (range 16–54) and marginal male predominance (*n* = 14, 58.3%). The presenting ECOG performance status (PS) was 1 in 13 (54.1%), 2/3 in 10 (41.6%) and 4 in 1 (4.1%) patients, respectively. The most common baseline symptoms were chest pain (*n* = 11, 45.8%), dyspnea (*n* = 10, 41.6%) and cough (*n* = 7, 29.1%). The distribution of stages at baseline diagnosis included 8 (33.3%) localised, 10 (41.6%) unresectable locally advanced and 6 (25%) metastatic diseases. At the time of data analysis for this study, this distribution comprised 3 (12.5%) localised, 5 (20.8%) locally advanced and 16 (66.6%) metastatic disease, respectively. 3 (12.5%) patients were misdiagnosed as lung cancers at peripheral centres during the initial evaluation, but were later found to have PTSS upon presenting to us.

The baseline median tumour size was 10 cm (range 2–20). The most common primary locations included the lung (*n* = 12, 50%), mediastinum (*n* = 6, 25%), chest wall (*n* = 4, 16.6%) and other sites such as cardiac SS in 1 (4.1%) patient. Among the 16 patients with metastatic disease, the common sites of metastases were lung (*n* = 10, 62.5%), pleural effusion (*n* = 4, 25%) and pleura (*n* = 3, 18.7%). 19 (79.1%) patients were positive for SS18 rearrangement by break-apart FISH while histopathologic tissue was not sufficient for molecular testing in the remainder. The clinico-pathological details of the study cohort are summarised in Table 1.

Curative management was planned based on MDT meetings between departments of medical oncology, surgical oncology and radiation oncology. Neoadjuvant chemotherapy (NACT) was administered in ten patients including 9 (90%) patients with locally advanced and 1 (10%) patient who had metastatic disease with a single site of metastases. Primary sites of disease managed with NACT were the lung (*n* = 5, 50%), mediastinum (*n* = 3, 30%) and chest wall (*n* = 2, 20%), with a median size of 12 cm (range 5–20). Indications for NACT decided by the MDT included cardiac and/or great vessel involvement (*n* = 4, 40%), extensive disease not amenable to complete surgery (*n* = 3, 30%), impending respiratory compromise (*n* = 2, 20%) and risk of nerve damage due to brachial plexus involvement (*n* = 1, 10%). NACT produced stable disease in 5 (50%), partial response in 3 (30%), complete response in 1 (10%) and local disease progression in 1 (10%) patients each. 16 (66.6%) patients underwent surgical resection, among whom 9 (56.2%) had upfront surgery and 7 (43.8%) were operated on after NACT. 11 (62.5%) had a complete resection (R0), 2 (18.7%) microscopically incomplete (R1) resection and 2 (12.5%) had the macroscopic residual disease (R2) (Table 2). 3 out of the 10 (30%) patients could not undergo surgery due to persistent extensive disease (*n* = 1, 33.3%), cardiac involvement (*n* = 1, 33.3%), negative consent by the patient due to the risk of brachial plexus damage (*n* = 1, 33.3%). RT was a part of the treatment regimen in 12 (50%) patients and 10 (41.6%) received postoperative RT while the remainder 2 (8.3%) received definitive RT for surgically unresectable disease.

Median 2 lines (range 1–6) of medical therapy (chemotherapy and/or targeted therapy) were given, including median 4 (range 3–6) cycles of neo(adjuvant) chemotherapy. 23 (95.8%) patients received anthracycline-based chemotherapy regimens in the first-line setting, either as (neo) adjuvant or palliative treatment. The most common medical regimens used in the 2nd line (*n* = 12, 50%) for advanced/metastatic disease included pazopanib (*n* = 5, 41.6%), gemcitabine with/without docetaxel (*n* = 4, 33.3%) and high dose ifosfamide (*n* = 2, 16.6%). Table 3 summarises the chemotherapy regimens used for the patients across all lines of treatment. Dexrazoxane was used prophylactically for cardio-protection in 2 (8.3%) patients. 1 patient who received dexrazoxane has been given postoperative mediastinal RT.

**Figure 1.**
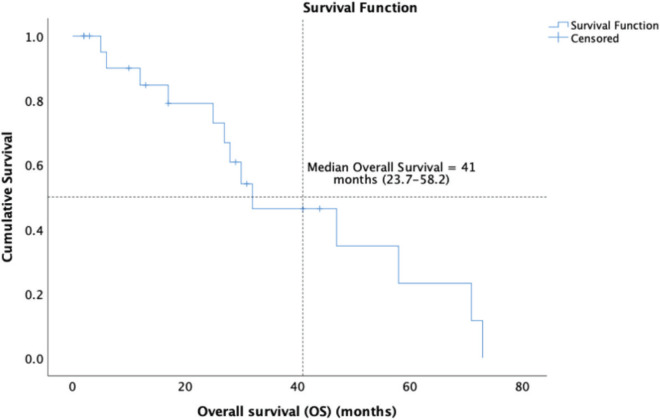
Kaplan-Meier analysis showing OS of patients with PTSS.

**Figure 2.**
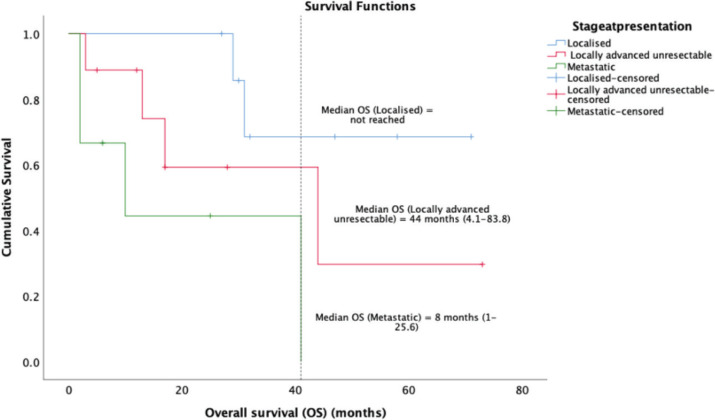
Kaplan-Meier analysis showing OS of patients with PTSS classified as per stage of disease at presentation.

**Figure 3.**
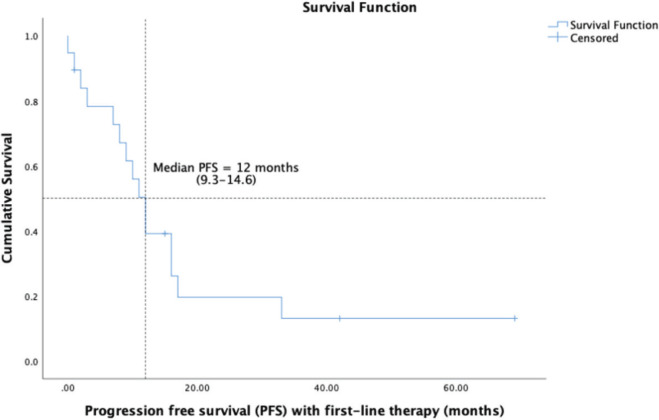
Kaplan-Meier analysis showing PFS of patients with PTSS.

14 (58.3%) patients of the cohort are currently alive, including seven living with metastatic disease. At a median follow-up of 32 months (range 16.7–47.2), the median OS was 41 months (95% CI: 23.7–58.2) and the 5-year OS was 37% (95% CI: 9.5%–64.4%) (Figure 1). The median OS as per disease stage was not reached for localised disease, 44 months (95% CI: 4.1–83.8) for locally advanced and 8 months (95% CI: 1–25.6) for metastatic disease (Figure 2). The median PFS with first-line of therapy was 12 months (95% CI: 10.5–13.4) (Figure 3), while it was 3 months (95% CI: 1.4–4.5) and 7 months (3.6–10.4) with second and third-lines, respectively (Table 3).

Metastatic disease at presentation (*p* = 0.01) and treatment with surgical resection (*p* = 0.005) (*p* = 0.005) were significantly associated with OS per univariate analysis but not by multivariate analysis (Table 4). Age, gender, ECOG PS (0 to 1 versus 2 and above), primary site (pulmonary versus non-pulmonary) and lymph node involvement were not found to be significantly associated with OS. Among the patients who underwent surgery, resection status, administration of adjuvant chemotherapy or adjuvant RT were not found to be associated with OS.

## Discussion

This is one of the largest contemporary studies covering all sub-sites of PTSS and its symptomatology, clinico-epidemiological profile, management and survival outcomes. Previous studies on primary thoracic sarcomas of all histologies have included patients with SS such as the analysis by Spraker *et al* [5] among 365 patients that included 43 patients with pulmonary SS. Studies that have included PTSS exclusively are few and with a limited number of patients due to the rarity of the condition (Table 5).

The demographic profile of our patients is concordant with that described in previous studies on SS wherein most patients are aged 40 years or less with an even gender distribution or a slight male predominance [3]. The clinical presentations in our patients depended on the primary site of involvement with a majority presenting with chest pain and respiratory complaints such as dyspnea and cough, as has been observed previously [6]. Thoracic sarcomas usually present in advanced stages due to their non-specific clinical presentations [7]. More than 40% of our patients had locally advanced unresectable disease at the time of baseline diagnosis and a median tumour size of 10 cm. This was also observed by Galetta *et al* [8] in their series on PTSS in which the tumours were similarly large with a median size of 8 cm.

The most reported primary sub-site in the previous studies on PTSS is pulmonary, which was also observed among our patients [6]. Molecular testing for translocation (X;18) assumes importance in the cases of PTSS with equivocal radio-pathological diagnoses [9]. The site of disease sometimes leads to constraints in obtaining tissue specimens for translocation testing. Despite this, we were able to perform molecular testing and demonstrate SS18 rearrangement in 79% of our patients. Extremely rare and aggressive sub-sites such as cardiac SS are included in our cohort, which has only been described in a few case reports and presents with a fulminant clinical course as in our patient [10].

Lung (41.6%), pleural effusion (16.6%) and pleura (12.5%) were the commonest sites of metastases in our series which have also been reported among patients with PTSS previously [11]. We observed some atypical metastatic sites, including lymph nodes (8.3%), brain (8.3%), breast (4.1%) and bone (4.1%). These rare sites were also reported among PTSS by Wang and Li [10] with nodal (9.9%), bone (2.5%) and brain (1.7%) metastases. A series on pulmonary sarcomas which had 12% patients with localised SS found a significant proportion of nodal metastases (16%) in pulmonary sarcomas [5]. This finding led the authors to opine that mediastinal nodal evaluation could be a routine part of the baseline evaluation of localised PTSS.

MDT is a key part of the management of sarcomas and is especially useful for PTSS which can be locally advanced and complex to treat. He *et al* [12] demonstrated a survival benefit among patients with PTSS who underwent MDT evaluation prior to initiation of treatment. Complete surgical resection in combination with RT and/or chemotherapy are the preferred treatment modalities. Among our patients, 66.6% underwent surgery as part of their treatment with 68.7% R0 resections. This is comparable to the proportion of R0 resections (63%) reported among primary pulmonary sarcomas in a previous series comprising of 22 patients [4].

The role of preoperative chemotherapy in PTSS remains poorly defined due to the lack of prospective data and the risk of selection bias in interpreting retrospective studies [13]. Among our patients (45.8%) who received anthracycline-based NACT, the objective response was a comparable 40% and delayed surgery was performed in 70% of patients with unresectable disease at baseline. Since PTSS tumours can be technically challenging to resect, administration of NACT can increase the number of complete surgeries.

**Figure 4.**
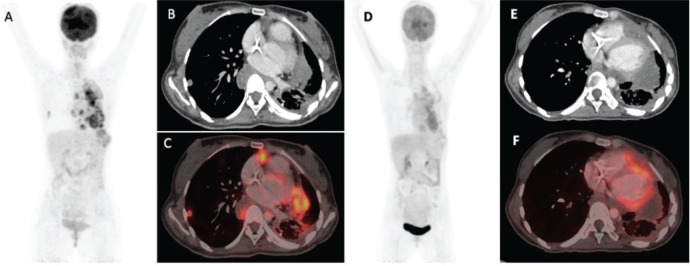
Fluorodeoxyglucose-positron emission tomography (PET) images of a patient with mediastinal SS treated with Anlotinib. (a): Pre-anlotinib maximum intensity projection (MIP), trans-axial computer tomography (CT) (b): And fused PET/CT (c): Images showing multiple variable sized lung subpleural and parenchymal nodules with some showing necrotic changes in bilateral lungs. (d–f): Post-anlotinib images showing decrease in size, uptake and number of most of the parenchymal and subpleural lesions in bilateral lungs with increase in associated necrotic component.

Anthracyclines and high-dose ifosfamide have shown efficacy in advanced SS with response rates varying from 25% to 60% in the first-line setting [14–17]. Our patients with advanced disease not previously exposed to anthracyclines were managed with single-agent doxorubicin, except for 1 patient who received ifosfamide. Agents that have shown anti-tumour activity in later lines in SS include trabectedin [18], pazopanib [19], regorafenib [20] and anlotinib [21] while immunotherapy and EZH2 inhibitors have produced dismal results [13, 22]. Among our patients, both cytotoxic chemotherapy and targeted agents were used in equal numbers including the first reported use of anlotinib (Figure 4) in PTSS so far. The use of dexrazoxane with anthracyclines among patients with sarcomas is currently inconsistent despite the demonstrated reduction in risk of clinical and subclinical chemotherapy-related cardiac adverse effects [23]. Dexrazoxane could be used with anthracyclines in PTSS, especially left-sided tumours, as was done in our patient who was planned for adjuvant mediastinal RT.

The survival outcomes of PTSS have been demonstrated to be poorer than SS (including all sub-sites) at a 5-year OS of 22% [24] in the former compared to 67% in the latter [25]. This was also observed by Lan *et al* [26] among pleuropulmonary and mediastinal SS where the median OS was 14.5 months in comparison to that reported in extremity SS (52 months). The median OS of 41 months in our study compares favourably to prior reports, especially driven by localised and locally advanced stages. However, survival outcomes were dismal among our patients with metastatic PTSS, emphasising the poor responses to palliative treatments. The stage of disease and surgical resection were the prognostic variables found significantly associated with OS on univariate analysis. The assessment for other variables could have been limited due to the small sample size of our cohort. In previous analyses, the prognostic variables associated with OS include ECOG PS ≥2, tumour size greater than 5 cm, incomplete surgical resection, non-administration of adjuvant chemotherapy and poor response to chemotherapy [8, 24, 26].

The limitations of our study include the constraints associated with its retrospective design. Also, a larger number of subjects would allow for a robust assessment of prognostic variables having an impact on the survival of patients with PTSS. However, our single-centre insight on this ultra-rare disease contributes to the paucity of currently reported literature.

## Conclusion

PTSS is a rare subset of thoracic malignancies that affects young individuals and presents with respiratory symptoms, large tumour size and advanced stage. The thoracic location poses challenges in terms of diagnosis and definitive management. Patients managed at non-expert centres may be misdiagnosed, leading to delayed or inadequate treatment. Multidisciplinary care and better palliative treatment options are required for the management of PTSS.

## List of abbreviations

CI, Confidence interval; DFS, Disease-free survival; ECOG, Eastern Cooperative Oncology Group; MDT, Multidisciplinary treatment; OS, Overall survival; PTSS, Primary thoracic synovial sarcoma; SS, Synovial sarcoma.

## Conflicts of interest

The authors have no conflicts of interest to declare.

## Funding

The authors have not received any financial support for the conduct of this study.

## Ethical approval

Ethical approval for the study has been obtained from the Institution Review Board of the All India Institute of Medical Sciences (IEC-678/01.10.2021).

## Figures and Tables

**Table 1.** Clinical details of patients with PTSS.

	Details: *n* (%)
Age distribution	Less than 18 years: 1 (4.1%) 18–60 years: 23 (95.9%)
Gender distribution	Male: 14 (58.3%) Female: 10 (41.7%)
ECOG PS	0–1: 13 (54.1%) 2–3: 9 (37.5%) 4: 1 (4.1%) Not available: 1 (4.1%)
Stage at baseline	Localised: 8 (33.3%) Locally advanced: 10 (41.6%) Metastatic: 6 (25%)
SS18 rearrangement	Positive: 17 (70.8%) Tissue not sufficient for testing: 7 (29.2%)
Symptoms	Chest pain: 11 (45.8%) Dyspnea: 10 (41.6%) Cough: 8 (33.3%) Haemoptysis: 3 (12.5%) Mass: 2 (8.3%) Fever: 1 (4.1%) Weight loss: 1 (4.1%) Jaundice: 1 (4.1%) Hemiparesis: 1 (4.1%) Back pain: 1 (4.1%)
Primary site	Lung: 12 (50%) Mediastinum: 6 (25%) Chest wall: 4 (16.6%) Pleura: 1 (4.1%) Heart: 1 (4.1%)
Metastatic site(s)	Lung: 10 (41.6%) Pleural effusion: 4 (16.6%) Pleura: 3 (12.5%) Lymph node: 2 (8.3%) Brain: 2 (8.3%) Liver: 1 (4.1%) Bone: 1 (4.1%) Breast: 1 (4.1%)

PTSS: Primary thoracic synovial sarcoma

**Table 2.** Details of curative therapy received by patients with PTSS.

	Details: *n* (%)
Types of definitive surgery	Total = 16 Lobectomy: 7 (43.7%) Wide local excision: 7 (43.7%) Pneumonectomy: 1 (6.2%) Debulking surgery by pericardial resection: 1 (6.2%)
Types of surgery for recurrent disease	Total = 6 Salvage local surgery: 3 Metastatectomy: 3
Types of curative RT	Total = 14 Adjuvant: 11 (84.6%) Radical (in unresectable disease): 3 (21.4%)

PTSS: Primary thoracic synovial sarcoma

**Table 3.** Details of medical treatment received by patients with PTSS.

	Details: *n* (%)	Median PFS (95% confidence interval)
First-line medical treatment	Total = 21 (1) Curative Ifosfamide-doxorubicin: 17 (80.9%) (2) Palliative Doxorubicin: 3 (14.2%) High-dose ifosfamide: 1 (4.7%)	12 (9.3–14.6)
Second-line medical treatment	Total = 12 Pazopanib: 5 (41.6%) Gemcitabine docetaxel: 3 (25%) High-dose ifosfamide: 2 (16.6%) Doxorubicin: 1 (8.3%) Gemcitabine: 1 (8.3%)	3 (1.4–4.5)
Third-line medical treatment	Total = 8 Pazopanib: 3 (37.5%) Trabectedin: 3 (37.5%) High-dose ifosfamide: 1 (12.5%) Regorafenib: 1 (12.5%)	7 (3.6–10.4)
Fourth-line medical treatment	Total = 4 Regorafenib: 1 (25%) Anlotinib: 1 (25%) Pazopanib: 1 (25%) Trabectedin: 1 (25%)	3 (1.0–4.9)

PTSS: Primary thoracic synovial sarcoma

**Table 4.** Cox-regression analysis of factors associated with OS in patients with PTSS.

	OS	
Variable	HR (95%CI)	*p* value
Gender 1. Male 2. Female	0.81 (0.23–2.91)	0.75
Age 1. Less than 30 years 2. Greater than 30 years	1.48 (0.42–5.24)	0.53
Presence of metastases at baseline 1. Yes 2. No	0.18 (0.04–0.69)	0.01
ECOG PS 1. 0–1 2. More than 2	1.35 (0.37–4.9)	0.65
Size of tumour 1. Less than 10 cm 2. Greater than 10 cm	0.41 (0.09–1.79)	0.24
Primary site 1. Pulmonary 2. Non-pulmonary	1.07 (0.29–3.96)	0.91
Lymph node involvement 1. Positive 2. Negative	2.27 (0.48–10.63)	0.29
Surgery performed 1. Yes 2. No	6.60 (1.79–24.39)	0.005
Surgical resection status 1. Complete microscopic resection 2. Microscopic/gross residual disease	1.17 (0.39–3.56)	0.77
Administration of RT 1. Yes 2. No	1.26 (0.50–3.17)	0.61
Administration of adjuvant chemotherapy 1. Yes 2. No	1.46 (0.28–7.39)	0.63

PTSS: Primary thoracic synovial sarcoma, OS: Overall Survival, ECOG PS: Eastern

Co-operative Oncology Group Performance Status

**Table 5.** Summary of previous published case series on PTSS.

	Authors (year of publication)	Sample size	Primary site(s)	SS18 rearrangement	Surgical details	Chemotherapy details	Survival outcome
1.	Zeren *et al* [27] (1995)	25	Pulmonary	Details NA	Surgery performe*d* = 19 Details NA = 6	Details NA	16% alive with disease; 16% alive without disease at 2–20 years
2.	Essary *et al* [6] (2001)	12	Pleuropulmonary	Positive = 3	Surgery performe*d* = 11 Details NA = 1	ACT = 3	2.5-year OS = 58%
3.	Duran-Mendicuti *et al* [28] (2003)	5	Chest wall Pulmonary	Positive = 4 Untested = 1	Surgery = 5	Details NA	Median OS = 22 months
4.	Okamoto *et al* [29] (2004)	11	Pulmonary	Positive = 11	Surgery = 10 Details NA = 1	Adjuvant chemotherapy = 2 Details NA = 1	50% death in 1–9 years
5.	Bégueret *et al* [9] (2005)	40	Intrathoracic	Positive = 39 Details NA = 1	Surgery = 33 Details NA = 4	Chemotherapy +/- RT = 3	Median DFS = 43 months
6.	Suster and Moran [30] (2005)	15	Mediastinal	Details NA	Surgery = 12 RT = 3	Details NA	80% recurrence at 1–3 years
7.	Hartel *et al* [31] (2007)	60		Positive = 36	Surgery = 41 Open biopsy = 14	Chemotherapy +/- RT = 4	Median DFS = 17 months
8.	Galetta *et al* [8] (2007)	15	Pulmonary Mediastinal	Positive = 15	Surgery = 15	ACT = 11 CTRT = 7 RT = 1	Median DFS = 15 months
9.	Kim *et al* [32] (2015)	14	Pulmonary	Positive = 6	Surgery = 8	Chemotherapy +/- RT = 7	2-year DFS 35.7%
10.	Lan et al [26] (2016)	26	Pleuropulmonary Mediastinal	Positive = 26	Surgery = 20	ACT = 7	Median DSS = 14.5 months
11.	Terra *et al* [33] (2018)	21	Mediastinal	Positive = 21	Surgery = 9 Details NA = 11	Chemotherapy +/- RT = 1	24% alive with disease at 6–45 months
12.	He *et al* [12] (2021)	13	Pleuropulmonary Mediastinal	Positive = 13	Surgery = 10	Chemotherapy +/- RT = 3	5-year OS = 30%
13.	Pieropan *et al* [24] (2022)	20	Pleuropulmonary Mediastinal Chest wall Trachea	Positive = 17 Tissue not sufficient= 3	Surgery = 20	NACT = 13 ACT = 2 RT = 11	5-year OS = 22% Median DFS= 8.5 months Median OS = 25 months
14.	Wang *et al* [7] (2022)	132 (12 institutional 121 SEER database)	Pulmonary		Surgery = 89	RT = 34 ACT = 8	5-year OS = 29% 5-year DSS = 31%

PTSS: Primary thoracic synovial sarcoma NA: Not available, ACT: Adjuvant chemotherapy, NACT: Neoadjuvant chemotherapy, RT: Radiotherapy, CTRT: Chemoradiotherapy, SEER: Surveillance, Epidemiology and End Results, OS: Overall Survival, DFS: Disease-free survival
